# IL-17A in the Psoriatic Patients' Serum and Plaque Scales as Potential Marker of the Diseases Severity and Obesity

**DOI:** 10.1155/2020/7420823

**Published:** 2020-06-05

**Authors:** Anna Michalak-Stoma, Joanna Bartosińska, Małgorzata Kowal, Dorota Raczkiewicz, Dorota Krasowska, Grażyna Chodorowska

**Affiliations:** ^1^Chair and Department of Dermatology, Venereology and Pediatric Dermatology, Medical University of Lublin, ul. Staszica 16, 20-081 Lublin, Poland; ^2^Institute of Statistics and Demography, Warsaw School of Economics, al. Niepodległości 162, 02-554 Warsaw, Poland

## Abstract

The aim of the study was to evaluate concentrations of IL-17 in the serum and plaque scales of psoriatic patients. We analyzed their association with the clinical activity of the disease and with body mass index (BMI). Demographic data, medical history, serum, and scale from psoriatic plaques for assessment of IL-17 were collected from all the participants. The disease severity was assessed with PASI (Psoriasis Area and Severity Index), BSA (Body Surface Area), PGA (Physician Global Assessment), NAPSI (Nail Psoriasis Severity Index), and DLQI (Dermatology Quality of Life Index) scores. Obesity was diagnosed by calculating body mass index. Serum and scale concentration of IL-17 was determined with Human IL-17A High Sensitivity ELISA kit and Human IL-17 ELISA kit. In the psoriatic patients, BMI was statistically significantly higher than in the control group. Most of the patients presented BMI higher than normal. Our study confirms that overweight is a problem among psoriatic patients. A significant positive correlation between the IL-17 serum and scale concentrations and psoriasis severity indicates that IL-17 can be used as the marker of disease severity. More data from human studies can be crucial for understanding that relationship between IL-17, psoriasis, and obesity.

## 1. Introduction

Psoriasis is a common chronic disease with the prevalence from 0.91 in the USA to 8.5% in Norway, whose new pathogenesis aspects are constantly being explored [1, 2]. Nowadays, psoriasis can be considered as an autoimmune and autoinflammatory disorder. It is already recognized that psoriasis is not only skin specific but also a systemic disease, and many comorbidities are found as psoriasis related [3]. The common diseases among psoriatic patients are psoriatic arthritis, diabetes mellitus, hypertension, hyperlipidemia, coronary heart disease, Crohn's disease, nonalcoholic fatty liver disease, T-cell lymphoma, and depression [4]. In addition, the rate of obesity for psoriatic patients is higher as compared with controls [5].

Since 2005, when Th17, the new subtype of T helper cells was discovered, many studies are conducted to find the role of IL-17 in psoriasis and other diseases' pathogenesis [6–9]. IL-17 (IL-17A) is a member of the cytokine family comprising IL-17A, IL-17B, IL-17C, IL-17D, IL-17E (IL-25), and IL-17F. Mostly, T helper 17 (Th17) cells secrete IL-17, but also, T regulatory (Treg) cells, NK cells, mast cells, and neutrophils are engaged in IL-17 production [10, 11]. IL-17 has a proinflammatory activity inducing the expression of proinflammatory cytokines, colony-stimulating factors, and chemokines from dendritic cells, neutrophils, T cells, monocyte/macrophages, and epithelial cells [6, 12, 13]. Some authors indicate the proinflammatory role of IL-17 in obesity [7, 14–16]. In obese people, IL-6 is overproduced by adipocytes and macrophages in visceral adipose tissue [17], what influences on IL-17 production. There is a positive feedback between IL-17 and IL-6, and IL-17 stimulates IL-6 production. On the other hand, there is evidence of the inhibitory effect of IL-17 on adipogenesis [14].

The aim of the study was to evaluate concentrations of IL-17 in the serum and plaque scales of psoriatic patients. We analyzed their association with the clinical activity of the disease and with body mass index (BMI).

### 1.1. Clinical Significances

- Psoriasis can be considered as an autoimmune and autoinflammatory disorder
- The rate of obesity for psoriatic patients is higher as compared with controls
- IL-17 has an important role in the pathogenesis of psoriasis
- IL-17 may have a proinflammatory role in obesity
- There is evidence of the inhibitory effect of IL-17 on adipogenesis
- Assessment of different biomarkers, for example, IL-17 in the plaque scale, can expand our knowledge about psoriasis pathophysiology
- Correlations between IL-17 concentration in serum and plaque scale of psoriatic patients, and disease severity and body mass index can be helpful in understanding the mechanisms of overweight and obesity among psoriatic patients

## 2. Materials and Methods

### 2.1. Studied Group

The study was conducted in patients hospitalized in the Department of Dermatology, Venereology and Pediatric Dermatology, Medical University of Lublin, Poland, due to psoriasis exacerbation.

The study comprised 21 male psoriatic patients and 20 male healthy controls. Inclusion criteria were the duration of psoriasis for at least one year, the presence of active psoriatic skin lesions with scale on the skin, and age above 18 years old. Patients who received any systemic treatment for psoriasis or applied steroids, retinoids, anthralin, or vitamin D analogs on the skin within the last 2 months were excluded from the study. Patients with the presence of any disease which required continuous treatment were excluded from the study.

Demographic data, medical history, serum, and scale from psoriatic plaques for assessment of IL-17 were collected from all the participants.

The study was approved by the Local Ethics Committee at the Medical University of Lublin (KE-0254/81/2015). The written informed consent for study participation was obtained from all study subjects.

### 2.2. Assessment of Psoriasis Severity and Obesity

The skin changes severity was assessed with the use of PASI (Psoriasis Area and Severity Index), BSA (Body Surface Area), and PGA (Physician Global Assessment) scores. Nail involvement was assessed with Nail Psoriasis Severity Index (NAPSI). The patients filled up the Dermatology Quality of Life Index (DLQI) form.

Obesity was diagnosed by calculating body mass index (BMI), which is the weight in kilograms divided by the square of the height in meters. The World Health Organization classifies BMI in adults between 18.5 and 24.9 as normal, 25-29 as overweight, and BMI 30 and more as obesity [5].

### 2.3. Assessment of IL-17 Serum Concentrations in the Psoriatic Patients and Controls

Blood samples were collected from psoriatic patients and controls and were centrifuged for 15 minutes at 1000 x g. Then, serum samples were subdivided into small aliquots to be stored at -80°C until tested for cytokine levels. In the studied psoriatic patients as well as the control group, concentrations of IL-17 were determined with the use of Human IL-17A High Sensitivity ELISA kit (eBioscience, Vienna, Austria), according to the manufacturer's instructions.

### 2.4. Assessment of IL-17 Scale Concentrations in the Psoriatic Patients

All patients were treated initially with a topical application of 5% salicylic acid ointment for desquamation. Plaque scales were collected from the most representative lesions. The same amount of scale from each patient was weighed. Then, plaque scales were suspended in phosphate-buffered saline (PBS) without calcium and magnesium and disrupted with Sonics Vibracell VCX130 in impulsing mode. Cell suspension was centrifuged for 10 minutes at 5000 x g. Then, scale samples were subdivided into small aliquots to be stored at -80°C until tested for cytokine levels. The concentration of IL-17 in the patient plaque scales was determined with the use of Human IL-17A High Sensitivity ELISA kit (eBioscience, Vienna, Austria) and Human IL-17 ELISA kit (R&D Systems, Minneapolis, MN, USA), according to the manufacturers' instructions. With Human IL-17A High Sensitivity ELISA kit, we have reached very high results, which were out of range. That is why we repeated the analysis with Human IL-17 ELISA kit (R&D Systems, Minneapolis, MN, USA).

### 2.5. Statistical Analysis

Statistical analysis was performed using STATISTICA software. Mean values (*M*) and standard deviations (SD) were calculated for continuous variables, or absolute numbers (*n*) and relative numbers (%) of occurrence of items of categorical variables. The following tests were applied: stochastic independence *χ*^2^, U Mann–Whitney test, *r* Pearson correlation coefficient. In all statistical tests, the level of significance was set at 0.05.

## 3. Results

### 3.1. Characteristics of the Studied Group

The characteristic of the studied groups is presented in Table 1. In the psoriatic patients, BMI was higher (27.32 ± 5.3) than in the control group (24.81 ± 3.09), and it was a statistically significant difference *p* < 0.05. Most of our patients presented BMI higher than normal: 8 (38%) patients were classified as overweight (range of BMI 25.0-29.9), and 8 (38%) patients were classified as obese (BMI above 30). Among the study group, 5 (24%) patients had normal BMI in the range 18.5-24.9. There was not any correlation between BMI and PASI, BSA, or PGA.

### 3.2. Serum and Scale Concentration of IL-17

Although serum IL-17 concentrations in psoriatic patients (4.24 ± 3.69 pg/ml) were higher than in the control group (3.06 ± 1.19 pg/ml), no significant differences were found (*p* > 0.05). The higher level of IL-17 was detected in scale from psoriatic plaques (68.32 ± 51.68 pg/ml) comparing to IL-17 serum level (4.24 ± 3.69 pg/ml). The relationship between the IL-17 serum and scale concentration was not statistically significant (*r* = 0.410, *p* = 0.065). A significant positive correlation between the IL-17 serum concentrations and psoriasis severity measured by the PASI (*r* = 0.61; *p* < 0.05), BSA (*r* = 0.608; *p* < 0.05), and PGA (*r* = 0.542; *p* < 0.05) was detected. A significant positive correlation between the IL-17 scale concentrations and psoriasis severity measured by BSA (*r* = 0.436; *p* < 0.05) was observed. In patients with higher BMI, the scale concentration of IL-17A was decreased (*r* = −0.482; *p* = 0.028). There were no statistical correlations between BMI and IL-17 serum level in psoriatic patient (*r* = −0.186, *p* = 0.435) as well as in the control group (*r* = 0.213, *p* = 0.367).

**Figure 1:**
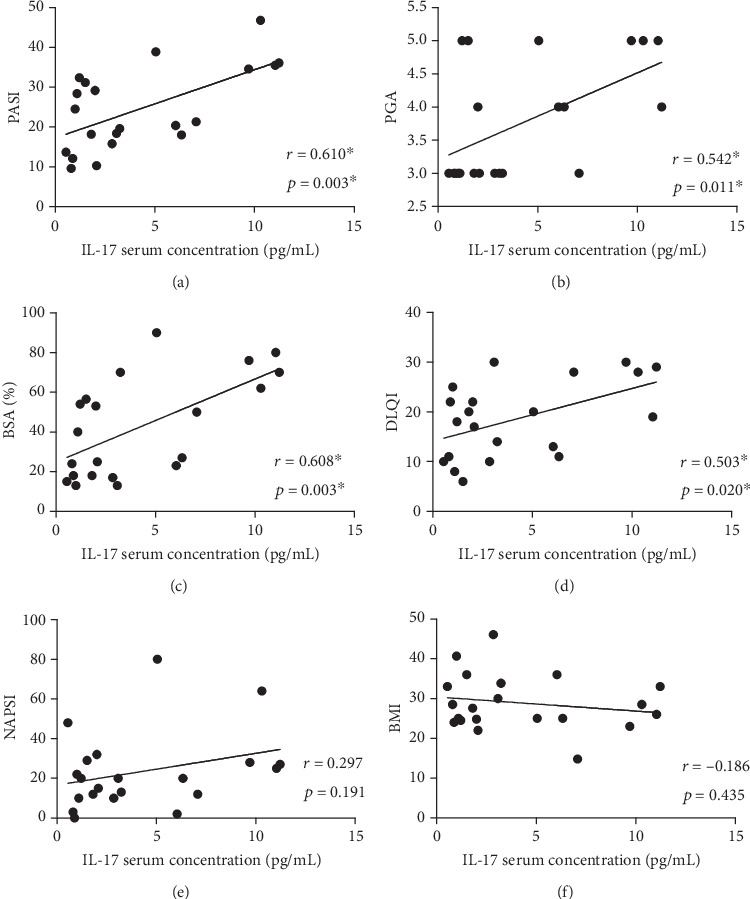
The correlation between IL-17 serum concentration and clinical features and disease severity in psoriatic patients; ∗ statistically significant correlation.

**Figure 2:**
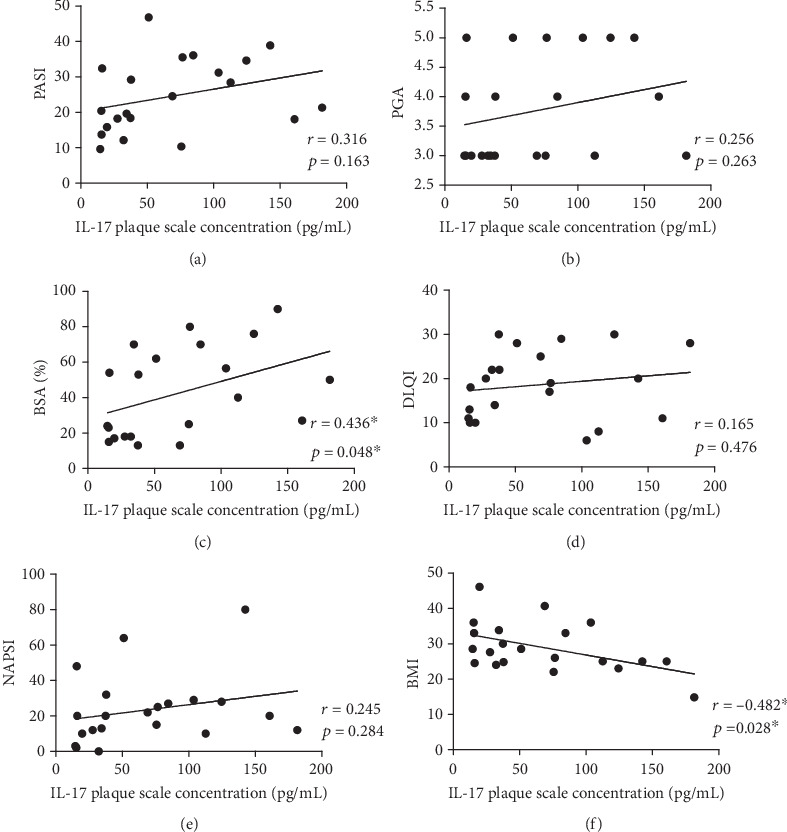
The correlation between IL-17 plaque scale concentration and clinical features and disease severity in psoriatic patients; ∗ statistically significant correlation.

All the correlations are presented in Table 2 and on the dot graphs (Figures 1 and 2).

## 4. Discussion

IL-17A is a very important cytokine in sustaining inflammation in psoriatic plaques. It influences the recruitment of inflammatory cells, enhances keratinocyte proliferation, and inhibits keratinocyte differentiation [18]. IL-17A is undetectable in normal skin; however, it was detected in skin lesions in allergic contact dermatitis and psoriasis vulgaris [9, 19–27].

Statistically significant differences in serum IL-17A level have been found in psoriatic patients comparing to healthy controls in some studies [28–33]. We found that serum IL-17A level was slightly higher in psoriatic patients than controls, although the result was not significant. Arican et al. and Choe et al. presented similar results [34, 35]. Choe et al. compared IL-17A serum level in two groups: the eruptive inflammatory (EI) group, included patients with quickly spreading guttate morphology and onset or reactivation of lesions shorter than 4 weeks, and the chronic stable (CS) group, included patients with large plaque morphology, an onset longer than 6 months and a stable clinical course of longer than 1 month. IL-17A showed higher levels in the EI group. Authors suggested that IL-17A is associated with EI psoriasis that has a recent onset, is quickly spreading, shows guttate morphology, and is often associated with severe pruritus and pustule formation [35]. Our patients had severe psoriasis with PASI from 10 to 47 lasting longer than 5 years. It could be the reason why IL-17A serum level was not significantly higher in psoriatic patients comparing to the control group.

Kyriakou et al. found that the median serum levels of IL-17 presented no significant difference between psoriatic patients with and without nail involvement. Target NAPSI score was not significantly correlated with the serum levels of IL-17 [36]. We did not observe a significant correlation between serum level of IL-17A and NAPSI either.

In our study, IL-17A serum levels correlated with PASI, BSA, and PGA. Other authors observed correlations of IL-17A with psoriasis severity [28, 33–35]. However, this correlation was not found in some papers [30]. Choe et al. noticed a linear correlation between serum cytokine level of IL-17A and the PASI in the CS group. In the IE group, the correlation was not observed [35].

In the PubMed database, we found a few studies which have been conducted to evaluate and compare the levels of epidermal growth factor (EGF) and the soluble EGF receptor, vascular endothelial growth factor (VEGF), interleukin 18 (IL-18), transforming growth factor beta1 (TGF-beta1), IL-4, IL-6, IL-10, IL-13, IL-1beta, IL-1ra, IP-10, MCP-1, MIP-1alpha, MIP-1beta, PDGF, and TNF-alpha in the serum and plaque scales of psoriatic patients [37–40]. However, to our knowledge, no study has ever been conducted to assess and compare the serum level of IL-17A in serum and plaque scale in psoriatic patients. In our study, the correlation between the IL-17 serum and scale concentration was not statistically significant. However, the level of IL-17 was higher in plaque scale than in serum. This can approve the local involvement of IL-17 in psoriatic plaque and scale formation. It was found that in the psoriatic lesions not only Th17 cells but also neutrophils and mast cells can produce IL-17 [41–44], which is why the level of IL-17 in the skin can be higher than in serum. Anti-IL-17 therapy is a very effective therapy, and it was observed that neutrophil-derived IL-17 is an early target of IL-17-directed therapies such as secukinumab [45]. A significant positive correlation between the IL-17 scale concentrations and psoriasis severity measured by BSA was observed. Our observations suggest that the concentration measurement of some cytokines, involved in the process of psoriatic lesion formation, can be performed in the psoriatic scale that is easy to obtain from patients.

Most of our patients were classified as overweight (38%) patients or as obese (38%) patients. There were not any correlations between BMI and PASI, BSA, or PGA. Our data are similar to the results presented by Bardazzi et al. In that study, overweight patients were accounted for 40% of enrolled patients, and obesity was observed in 42% of patients [46]. They did not show statistically significant differences between PASI in selected groups of patients either. However, it was observed that in obese patients PASI was higher (32.36 ± 12.79) comparing to the normal and overweight patients with PASI 19.63 ± 9.17. We did not have similar observations. In the psoriatic patients, BMI was higher than in the control group, and it was a statistically significant difference. Many studies including very big numbers of psoriatic patients demonstrated that the risk of severe psoriasis in an overweight or obese population was higher than in normal-weight people [5, 47–50]. The meta-analysis of 16 observational studies including 201,831 patients with psoriasis demonstrated that compared with individuals from the general population, psoriatic patients, especially with severe psoriasis, are at significantly higher odds of obesity [5, 51]. However, according to current epidemiological evidence, it is impossible to establish which comes first, psoriasis or obesity. Evidence strongly suggests that obesity is an independent risk factor for psoriasis [5].

The main limitation of our study is the use of BMI for obesity assessment. BMI is used extensively in research and clinical practice; however, it could have limited diagnostic performance due to its inability to discriminate between fat and lean mass [52–56]. Whereas the body fat % (BF%) has been associated with some health problems like cardiovascular diseases [53]. BMI fails to indicate the severity of the comorbidities [54]. In the study of Romero-Corral A. et al., bioelectrical impedance was used to assess body fatness. They found that using BMI as a marker of obesity, they misclassified ≥50% of patients with excess body fat as being normal or just overweight [53]. In a recent study, Galluzzo et al. also indicated that bioelectrical impedance analysis is able to identify obese psoriatic patients in the group of normal-weight patients detected by using BMI alone and suggested screening for body fat distribution in psoriatic patients with a normal or slightly elevated BMI, since this might be crucial to identify patients at risk of obesity-related diseases, like hyperlipidemia, coronary artery disease, hypertension, and diabetes, and to enhance prevention strategies [52]. Galluzo et al. found also that the values of adipose tissue were not correlated with psoriasis severity, which is similar to our results of BMI/PASI correlations [52]. Other methods which can be helpful in BF% measurement are hydrostatic weighting, energy-dual X-ray absorptiometry, and air displacement plethysmography [53, 57]. We are going to perform an additional BF% evaluation in future studies.

The link between psoriasis and obesity can be explained with new knowledge about white adipose tissue which is an essential endocrine organ secreting a wide range of soluble mediators involved in immunity, inflammation, and metabolic and appetite regulation [5, 58, 59]. The soluble mediators possess proinflammatory actions and can contribute to the low-grade inflammatory state in obese individuals [58]. There are also studies suggesting that adiposity may lead to the induction of T-helper 17 cells (Th17) which take part in the involvement in the pathogenesis of autoimmune diseases including psoriasis [5, 60].

Correlations between IL-17, psoriasis, and increased adiposity were poorly investigated so far. Recent studies have observed that serum levels of IL-17 are significantly higher in obese subjects compared with controls. In particular, a study based on obese women underlines a role for IL-17 and IL-23 as potential markers of the inflammatory syndrome that characterizes obesity [61–63]. We did not observe any statistically significant correlation between IL-17A serum concentration and BMI. However, in patients with higher BMI, the scale concentration of IL-17A was decreased (*p* = 0.028, *r* = −0.482).

IL-17 belongs to a family of proinflammatory cytokines, and its role in the pathogenesis of obesity is as important as that of IL-1, IL-6, IFN, or TNF-alpha [17, 64]. Obesity positively correlated with both IL-17 expression and disease severity in IL-17-driven inflammatory mouse models [65, 66]. Pini and Fantuzzi demonstrated an increased concentration of IL-17A in obese mice in provoked acute inflammation [16]. Adipocytes and macrophages in visceral adipose tissue produce IL-6 which activates intracellular STAT3 and retinoid-related orphan receptors, increasing differentiation of the Th17 cells [17]. Recently, it was demonstrated that TGF-beta is engaged in the enhancement and mediation of this process [67]. An increased serum concentration of amyloid A in inflammation associated with obesity influences the differentiation of Th17 lines [17]. TNF-alpha production is also increased in obesity and correlates with the IL-17 production [68].

On the other hand, IL-17 inhibits adipogenesis, reduces lipid and glucose uptake acting on both preadipocytes and adipocytes [61, 69, 70]. IL-17 deficiency increases diet-induced obesity and accelerates adipose tissue accumulation [15]. IL-17 modifies also the expression of adipogenic transcription factors [15]. An increased IL-17 secretion results in increased activity of cyclooxygenase-2, therefore increased prostaglandin E2 production, and prostaglandin E2 inhibits differentiation of adipocytes. Increased IL-17 concentration is also responsible for lipolysis in adipocytes [64].

## 5. Conclusions

The group of male patients with severe psoriasis presented in this study has BMI higher than in the control group. 76% of patients presented BMI higher than normal. Our study, similar to other publications, confirms that overweight is a problem among psoriatic patients. The role of IL-17 in the psoriasis pathophysiology is recognized. A significant positive correlation between the IL-17 serum concentrations as well as IL-17 scale concentrations, and psoriasis severity indicates that IL-17 can be suggested as the marker of the disease severity. However, it remains an unresolved question about the influence of IL-17 on overweight and obesity in psoriatic patients. In patients with higher BMI, the scale concentration of IL-17A was decreased, which can be significant, but it could also be an unimportant result since the small group of patients. Studies should be extended to check this correlation. The studies on animal models suggest the importance of IL-17 in the obesity pathophysiology. That is why more data from human studies can be crucial for understanding the relationship between IL-17, psoriasis, and obesity.

## Figures and Tables

**Table 1:** Characteristics of studied groups.

Variable	Psoriatic patients				Control group			
	Min	Max	M	SD	Min	Max	M	SD
Age (years)	26	76	50.14	14.51	27	75	46.85	13.44
BMI	18.8	46.1	27.32	5.30	20.68	30.19	24.81	3.09
Age of psoriasis onset (years)	1	71	29.29	17.37				
Duration of psoriasis (years)	5	44	20.86	12.82				
PASI	10	47	24.52	10.36				
PGA	3	5	3.76	0.89				
BSA (%)	13	90	42.6	25.29				
NAPSI	0	80	23.43	19.82				
DLQI	6	30	18.62	7.76				
Il-17 serum concentration	0.54	11.23	4.24	3.69	1.21	6.23	3.06	1.19
IL-17 scale concentration	14.74	181.57	68.32	51.68				

**Table 2:** The correlation between IL-17 serum, scale concentration and clinical features and disease severity in psoriatic patients; ∗ statistically significant correlation.

Variable	IL-17 serum concentration		IL-17 scale concentration	
	*r*	*p*	*r*	*p*
Age	-0.413	0.063	-0.266	0.243
Age of psoriatic onset	-0.420	0.058	-0.064	0.782
Duration of psoriasis	0.103	0.657	-0.214	0.351
BMI	-0.186	0.435	-0.482∗	0.028∗
PASI	0.610∗	0.003∗	0.316	0.163
PGA	0.542∗	0.011∗	0.256	0.263
BSA	0.608∗	0.003∗	0.436∗	0.048∗
DLQI	0.503∗	0.020∗	0.165	0.476
NAPSI	0.297	0.191	0.245	0.284

## Data Availability

The data used to support the findings of this study are available from the corresponding author upon request.
